# A mendelian randomization study revealing that metabolic syndrome is causally related to renal failure

**DOI:** 10.3389/fendo.2024.1392466

**Published:** 2024-06-07

**Authors:** Xianfu Cai, Decai Wang, Jianjun Wang, Chenguang Ding, Yang Li, Jin Zheng, Wujun Xue

**Affiliations:** ^1^ Department of Renal Transplantation, the First Affiliated Hospital of Xi’an Jiaotong University, Xi’an, Shaanxi, China; ^2^ Department of Urology, Mianyang Hospital Affiliated to School of Medicine, University of Electronic Science and Technology of China, Mianyang Central Hospital, Mianyang, Sichuan, China; ^3^ Department of Hepatobiliary Surgery, Mianyang Hospital Affiliated to School of Medicine, University of Electronic Science and Technology of China, Mianyang Central Hospital, Mianyang, Sichuan, China

**Keywords:** renal failure, chronic kidney disease, end-stage renal disease, abdominal obesity, metabolic syndrome, mendelian randomization

## Abstract

**Background:**

The onset and progression of chronic kidney disease (CKD) has been linked to metabolic syndrome (MetS), with the results of recent observational studies supporting a potential link between renal failure and MetS. The causal nature of this relationship, however, remains uncertain. This study thus leveraged a Mendelian Randomization (MR) approach to probe the causal link of MetS with renal failure.

**Methods:**

A genetic database was initially used to identify SNPs associated with MetS and components thereof, after which causality was evaluated through the inverse variance weighted (IVW), MR-Egger regression, and weighted media techniques. Results were subsequently validated through sensitivity analyses.

**Results:**

IVW (OR = 1.48, 95% CI = 1.21–1.82, P =1.60E−04) and weighted median (OR = 1.58, 95% CI =1.15–2.17, *P* = 4.64E-03) analyses revealed that MetS was linked to an elevated risk of renal failure. When evaluating the specific components of MetS, waist circumference was found to be causally related to renal failure using the IVW (OR= 1.58, 95% CI = 1.39–1.81, *P* = 1.74e-11), MR-Egger (OR= 1.54, 95% CI = 1.03–2.29, *P* = 0.036), and weighted median (OR= 1.82, 95% CI = 1.48–2.24, *P* = 1.17e-8). The IVW method also revealed a causal association of hypertension with renal failure (OR= 1.95, 95% CI = 1.34–2.86, *P* = 5.42e-04), while renal failure was not causally related to fasting blood glucose, triglyceride levels, or HDL-C levels.

**Conclusion:**

These data offer further support for the existence of a causal association of MetS with kidney failure. It is thus vital that MetS be effectively managed in patients with CKD in clinical settings, particularly for patients with hypertension or a high waist circumference who are obese. Adequate interventions in these patient populations have the potential to prevent or delay the development of renal failure.

## 1 Introduction

Metabolic syndrome (MetS) has emerged as an increasingly pressing threat to global public health (1). It is defined as a series of abnormal conditions including hypertension, abdominal obesity, decreased HDL-C levels, elevated levels of triglycerides (TGs), and hyperglycemia. This metabolic dysregulation can ultimately cause substantial renal damage such that the kidneys are among the most well-established target organs associated with MetS. MetS is thus closely related to chronic kidney disease (CKD) onset and progression (2, 3). Despite several reports documenting a link between MetS and end-stage renal disease (ESRD) or renal failure, whether these links are causal in nature remains an open question (3, 4). The kidneys are highly vascularized such that they are highly susceptible to changes at the microvascular level. Causal links between MetS and the onset, acceleration, and progression of CKD have been reported. There is thus a vital need to fully elucidate the mechanisms through which MetS contributes to renal failure so that effective interventional strategies can be devised to slow the progression of disease and prevent ESRD (5).

Kidney disease impacts >850 million people worldwide, including acute kidney injuries, CKD, and treated kidney failure characterized by renal insufficiency with a GFR < 15 mL/min/1.73 m^2^ (6). The leading causes of renal disease include arterial hypertension and diabetes mellitus (7), with other contributing conditions also playing a role such as genetic mutations, renal vasculitis, infectious glomerulonephritis, ureteral obstruction, and autoimmune disorders including Goodpasture’s disease, lupus nephritis, and IgA nephropathy (8, 9). Given the immense public health impact of CKD, particularly in lower and low-middle income countries, it is vital that new strategies be developed to reduce the burden of disease (10). While prior studies have documented links between MetS and both CKD and ESRD (3, 11, 12), relatively little data is currently available that pertains specifically to the association between MetS and CKD or ESRD onset (13). Those studies that have been performed were largely retrospective in design such that they are susceptible to the potential for bias due to the effects of confounding factors including limited sample sizes, short follow-up durations, and reverse causality (14).

Mendelian randomization (MR) strategies provide a means of minimizing the effects of confounding factors or reverse causality, making them ideal for probing the causal link of MetS with renal failure (15). In the absence of pleiotropy, MetS-related genetic mutations should have an impact on renal failure incidence if there is indeed a causal link of the exposure (MetS) with the outcome variable (renal failure). Given the absence of any definitive conclusions regarding the causal link of MetS with renal failure, the present study was developed in which an MR analysis was performed to probe the causal impacts of MetS on renal failure in order to provide guidance for efforts to treat or prevent renal failure.

## 2 Methods

### 2.1 Overview of MR analyses

**Figure 1:**
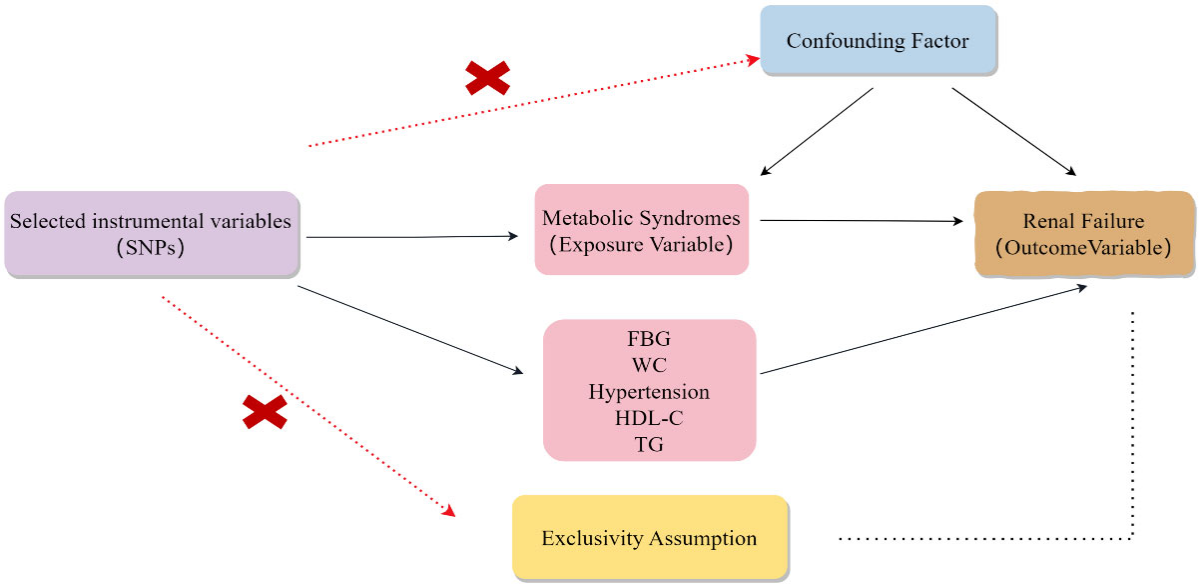
The hypotheses that must be met for Mendelian randomization studies.

MR analyses employ SNPs as instrumental variables (IVs), providing a means of overcoming the effects of confounding factors when examining potential causal links between particular risk factors and forms of disease (16). This analytical strategy takes advantage of the wealth of genetic variants present in genome-wide association study (GWAS) datasets to facilitate the identification of SNPs related to particular exposures (e.g., MetS) and outcomes (e.g., renal failure). As genotypes are randomly established via meiosis before birth (17), they can be utilized in MR analyses to mitigate possible confounding effects and to explore causal links of exposures with outcomes. IVs employed in these MR analyses must meet with three key assumptions (18, 19): they must be strongly correlated with the exposure being studies (20), independent of confounding factors, they must not be linked to the outcome through any other cause (21), Influencing the outcomes exclusively through the exposure, as illustrated in **Figure 1**.

### 2.2 Data source

The FinnGen project, a large genetics research program in Finland, compiles GWAS and PheWAS findings across various diseases to investigate the correlation between genomic data and health traits in the Finnish population. The GWAS ATLAS database encompasses GWAS outcomes from 4,756 human phenotypes, with genetic correlation analyses conducted among different traits. The GWAS Catalog curated by the European Bioinformatics Institute (EBI) summarizes published GWAS findings across a wide spectrum of diseases and phenotypes. CNCR CTGLAB offers GWAS summary statistics for over 100 common diseases and human phenotypes, spanning metabolic, cardiovascular, immune, oncological, and other disease categories. The data are sourced from public databases available from the aforementioned sources. Detailed URLs for data collection are provided in **Table 1**. All personal data were anonymized, and ethical approval and informed consent were waived.

**Table 1:** Study data sources and strength analyses.

Exposure	Ancestry	Sample size (case/control)	Data sources	R^2^ (%)	F-statistic	Source
Metabolic syndrome	European	461,920	Jorim J. Tielbeek et al.	0.98	54.37	https://ctg.cncr.nl/software/summary_statistics
FBG	European	13,556	Alisa K Manning et al	0.76	82.24	https://gwas.mrcieu.ac.uk/datasets/ebi-a-GCST005186/
WC	European	462,166	MRC-IEU	0.75	51.63	https://gwas.mrcieu.ac.uk/datasets/
Hypertension	European	119,731/343,202	MRC-IEU	0.69	56.77	https://gwas.mrcieu.ac.uk/
HDL-C	Mixed (96% European)	187,167	GLGC	0.90	119.70	https://gwas.mrcieu.ac.uk/datasets/
Triglycerides	Mixed (96% European)	177,861	GLGC	0.90	150.16	https://gwas.mrcieu.ac.uk/datasets/
Renal failure	European	5,951/212,841	FinnGen Consortium	/	/	www.finngen.fi/f

### 2.3 Instrumental variable selection

IVs were obtained from the most recent summary-level GWAS data and used to conduct a two-sample MR analysis investigating causal associations of MetS with renal failure. Variants for MetS were from the Complex Trait Genetics Lab (CTG) dataset comprising 461,920 individuals of European ancestry with structural equation modeling to identify MetS-related genetic variants (22). IVs were selected based on a strict series of criteria (19): only those SNPs exhibiting genome-wide significance (*P* < 5 × 10^-8^) were eligible for consideration (20), employing the PLINK algorithm with precision, the removal of linkage disequilibrium was undertaken by setting a stringent r^2^ threshold at 0.001, coupled with a clumping distance parameter of 10,000 kb (21), and eliminating SNPs related to confounders (23), as well as using harmonizing processes to remove ambiguous and palindromic SNPs. Pleiotropic SNPs were also removed. The MR analysis included 161 total SNPs. Details regarding the utilized IVs are presented in **Table 1**, with the F-statistic values > 10 being indicative of greater strength when predicting renal failure (24). Genetic variants related to MetS were derived from a GWAS dataset and screened to ensure the adequate strength of these variants as IVs. Only those IVs with an F-statistic > 10 were included, ensuring that the findings were robust (see **Table 1**). Genetic instruments pertaining to fasting blood glucose (FBG) could be attained from a comprehensive database encompassing 58,074 participants, all of European ancestry, adjusting for body mass index (BMI) (25), increasing the validity of these results. Screening led to the identification of 7 FBG-related SNPs that were included in this analysis. A comprehensive set of 537 SNPs was involved in the assay. Waist circumference (WC)-related genetic instruments were derived from the Medical Research Council Integrative Epidemiology Unit (MRC-IEU) UK Biobank GWAS pipeline consisting of data from 462,166 individuals of European ancestry. Data pertaining to hypertension from the MRC-IEU UK Biobank pipeline was based on results from 119,731 cases and 343,202 controls, all of whom were of European ancestry, yielding 301 SNPs following screening. Summary statistics for HDL-C and TG were attained from the Global Lipids Genetics Consortium (GLGC), encompassing a participant pool where 96% belonged to European ancestry (26). After screening, 122 and 70 SNPs associated with these two respective parameters were identified.

### 2.4 Statistical analysis

Bonferroni-corrected two-tailed *P*-values < 0.008 (0.05/6 = 0.008) were regarded as significant for this MR analysis. MR estimates are presented in the form of odds ratios (ORs) and 95% confidence intervals (CIs). R^2^ values were computed to indicate the proportion of exposure variance explained by the selected IVs. The strength of the relationships between IVs and exposures was assessed with the F-statistic (**Table 1**). It was attempted to carry out the statistical analysis utilizing the TwoSampleMR package (v 0.5.8) in R (v 4.3.2). To ensure the robustness of the MR estimates, we conducted additional sensitivity analyses. In our MR analysis, where all SNPs were assumed to be valid instrumental variables, the inverse variance weighting (IVW) method was our primary analytical approach. Furthermore, we employed three sensitivity analyses to validate our findings, including the weighted median method, the MR-Egger method, and the MRPRESSO (Multiple Residuals and Outliers of Effectiveness) method.

## 3 Results

### 3.1 Causal associations of MetS with renal failure

**Figure 2:**
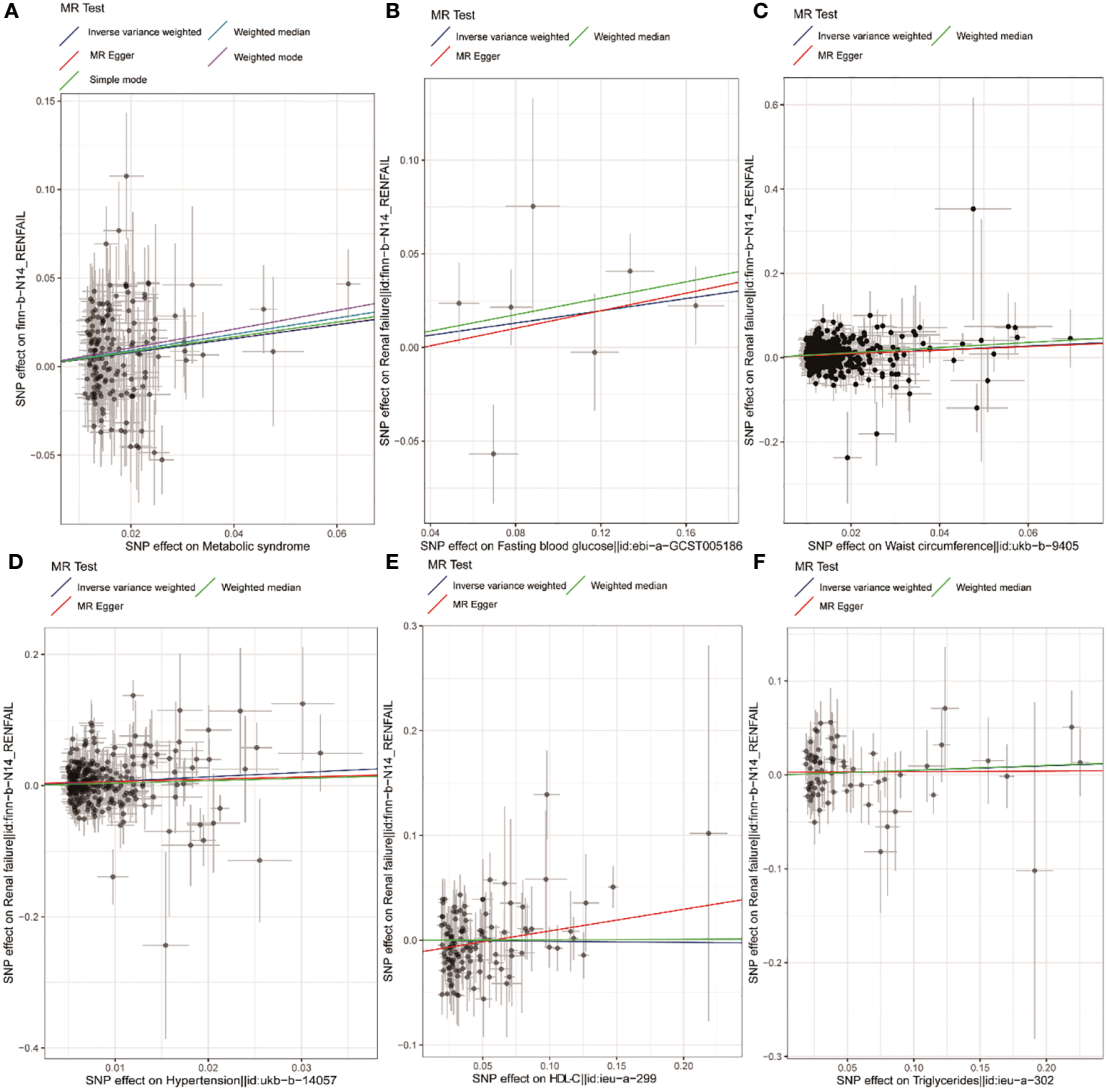
Scatter plots corresponding to MetS and components thereof. Scatter plots are shown corresponding to the relationship between renal failure and MetS **(A)**, FBG **(B)**, WC **(C)**, hypertension **(D)**, HDL-C **(E)**, and TG **(F)**. MetS, metabolic syndrome; FBG, fasting blood glucose; WC, waist circumference; HDL-C, high-density lipoprotein cholesterol; TG, triglycerides.

**Figure 3:**
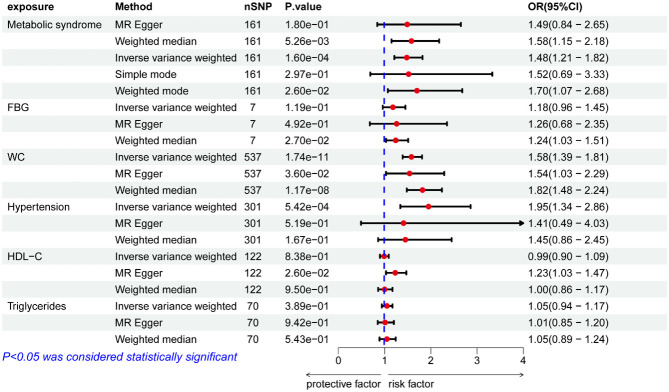
ORs pertaining to MetS and its constituent components.

Based on the PLINK algorithm clumping step, 161 SNPs intricately linked to MetS were selected for use in this MR analysis. SNPs related to the outcome and palindromic SNPs were excluded. The results of MR and sensitivity analyses are shown in **Table 2** while corresponding scatter plots and forest plots are presented in **Figures 2**, **3**. These plots confirmed a causal link of MetS with a greater risk of renal failure, as confirmed through the IVW (OR = 1.48, 95% CI = 1.21–1.82, *P* = 1.60E−04), weighted median (OR = 1.58, 95% CI = 1.15–2.17, *P* = 4.64E-03), and MR-Egger (MRE) analyses (OR = 1.54, 95% CI = 1.03–2.29, *P* = 0.044) (**Table 2**). These results remained significant when analyses were conducted with random-effects models owing to the presence of heterogeneity (*P* < 0.05) as demonstrated by the Cochran Q-test for MRE (*P* = 0.044) and IVW analyses (*P* = 0.049). No significant MRE intercept (MREI) values were detected (intercept = -5.04e-05; *P* = 0.992), and MR-PRESSO results (*P*-value = 0.748) did not reveal any directional pleiotropy. Symmetrical funnel plots were generated (**Supplementary Figure S1** (namely SFS1)), and leave-one-out (LOO) sensitivity analyses did not detect any individual SNPs that significantly affected the overall results (refer to **Supplementary Figure S2** (namely SFS2)). These results confirmed the stability of this MR analysis.

**Table 2:** The relationship between MetS or components thereof and renal failure in the MR analysis.

Exposure	Method	OR (95%CI)	*P*-value	Cochran Q test P-value	Egger_ intercept	*P*-Egger_ intercept	MR-PRESSO P-value
Metabolic syndrome							0.748
	Inverse variance	1.48 (1.21–1.82)	1.60E-04	0.049			
	MR Egger	1.49 (0.84–2.65)	1.80E-01	0.0442	-5.04e-05	0.992	
	Weighted median	1.58 (1.15–2.17)	4.64E-03				
FBG							0.232
	Inverse variance	1.18 (0.96–1.45)	0.119	0.131			
	MR Egger	1.26 (0.68–2.35)	0.492	0.083	-0.00849	0.819	
	Weighted median	1.24 (1.03–1.51)	0.027				
WC							0.05
	Inverse variance	1.58(1.39–1.81)	1.74E-11	0.048			
	MR Egger	1.54(1.03–2.29)	0.036	0.045	0.00050	0.870	
	Weighted median	1.82(1.48–2.24)	1.17E-08				
Hypertension							0.486
	Inverse variance	1.95(1.34–2.86)	0.001	9.56e-06			
	MR Egger	1.41 (0.49–4.03)	0.519	8.76e-06	0.00280	0.515	
	Weighted median	1.21 (0.86–2.45)	0.167				
HDL-C							0.056
	Inverse variance	0.99 (0.09–1.09)	0.838				
	MR Egger	1.23 (1.03–1.47)	0.026	0.146	-0.01185	0.006	
	Weighted median	1.00 (0.86–1.17)	0.950	0.066			
Triglycerides							0.495
	Inverse variance weighted	1.05(0.94–1.17)	0.389	0.470			
	MR Egger	1.01(0.85–1.20)	0.942	0.449	0.00270	0.549	
	Weighted median	1.05(0.89–1.25)	0.562				

### 3.2 Causal associations of MetS components with renal failure

Using IVs related to different exposures, including 7, 537, 301, 122, and 70 SNPs associated with FBG, WC, hypertension, HDL-C, and TG, the causal links between different components of MetS and renal failure were further probed. Following the exclusion of unavailable or palindromic SNPs from the pooled dataset, these analyses revealed that increased WC was causally related to renal failure when using the IVW, MRE, and weighted median (OR= 1.58, 95% CI = 1.39–1.81, *P* = 1.74e-11; OR= 1.54, 95% CI = 1.03–2.29, *P* = 0.036; OR= 1.82, 95% CI = 1.48–2.24, *P* = 1.17e-8). Both scatter and forest plots, as illuminated in **Figures 1** and **2**, provided visual evidence in support of these conclusions. The Cochran Q test revealed significant heterogeneity (IVW, *P* = 0.048; MRE, *P* = 0.045), such that a random-effects model was employed for these analyses. No multicollinearity was detected when using the MREI test (intercept = - 0.00050; *P* = 0.870) or the MR-PRESSO test (*P* value (particularly for global test) = 0.05). A funnel plot (SFS1) and the LOO approach (SFS2) additionally supported these findings. MR analyses performed with the IVW method also revealed a clear, significant, causal link between hypertension and an increased risk of renal failure (OR= 1.95, 95% CI = 1.34–2.86, *P* = 5.42E-04) (**Table 2**), with the results being presented in scatter and forest plots, as illustrated in **Figures 1**, **2**. Notably, a random-effects model was employed owing to the presence of significant heterogeneity (IVW, *P* = 9.56e-06; MRE, *P* = 8.76e-06). No multicollinearity was detected using the MREI test (intercept = - 0.00280; *P* = 0.515) or MR-PRESSO test (*P* value (particularly for global test) = 0.486). Renal failure was not significantly associated with any of the three other analyzed indices (**Table 2**).

## 4 Discussion

This study is the first report to our knowledge employing a large-scale MR approach to probe the potential causal association of MetS or components thereof with renal failure incidence. These results revealed a significant link between the development of renal failure and MetS, WC, and hypertension. The robust nature of these results was further confirmed through sensitivity and multiplicity analyses.

MetS consists of a series of cardiovascular risk factors that are related to the onset and progression of CKD (5), with recent evidence strongly linking MetS and CKD (27). In a cross-sectional analysis, MetS incidence was found to be related to CK incidence irrespective of BMI, while WC and hypertension were identified as the MetS components most closely related to renal failure (28), in line with the present results. A meta-analysis determined that the combination of WC and TF levels can be effectively used to assess CKD risk (29), and there is also cross-sectional evidence suggesting that MetS or chronic inflammation are related to renal impairment, with a particularly strong link between low-grade inflammation and stage 3+ CKD in individuals with MetS, whereas the same was not true in individuals without MetS (30). Transplant kidney loss and cardiovascular disease-related death have been linked to MetS diagnosis following renal transplantation (31). Further cross-sectional evidence suggests that obese individuals exhibit a significant correlation between MetS and CKD, while the same is not true in individuals who are non-obese (32). These discrepant results may be related to confounding factor-related biases introduced in observational studies. As the present MR analyses were conducted at the genetic level, this research could notably mitigate the influences of confounding variables, encompassing external environmental factors, lifestyle elements, and dietary practices, thereby boosting the reliability of the outcomes.

Our study findings indicate that there is no causal relationship between FBG, HDL-C, and triglycerides and renal failure in individuals with MetS. This lack of causality may be attributed to various factors, such as genetic predisposition, environmental influences, and other confounding variables. It is important to note that while hypertension, abnormal glucose metabolism, hyperuricemia, and dyslipidemia are established risk factors for CKD (33–35), they are not directly linked to renal failure in the context of MetS. Animal and human studies have demonstrated that hyperinsulinemia can enhance sodium reabsorption and induce glomerular hyperfiltration, thereby contributing to kidney injury (36–38). Excessive levels of insulin may result in renal damage. Additionally, insulin resistance can initiate and exacerbate CKD by impairing podocytes and basement membranes through chronic inflammation and oxidative stress. Despite these theoretical underpinnings, abnormalities in glucose metabolism and lipid metabolism have the potential to serve as risk factors for CKD and kidney failure. Metabolic markers such as (Triglyceride-glucose)TyG, MetS-IR, and TG/HDL-C have been observed to be markedly increased in individuals with metabolic syndrome. However, in certain cross-sectional studies, elevated TyG levels were linked to CKD solely in hypertensive patients aged over 65 years (39). One study revealed a notable association between the TyG index and CKD in hypertensive patients with abnormal glucose metabolism, independent of other conventional risk factors. In contrast, a nonlinear relationship was observed between the TyG index and CKD, where the risk initially decreased and then increased with a gradual elevation in TyG levels (40). This pattern mirrors the findings of our study, suggesting a potential strong causal relationship between these risk factors in stratified patient groups. There could be unaccounted genetic variants, treatment interventions, lifestyle factors, and disease stratification variables that might influence the causal association between components of Mets and kidney failure.

As the anthropometric parameters that can most effectively measure visceral adipose tissue mass, WC is strongly linearly correlated with abdominal obesity (41). Obesity, in turn, is an important metabolic condition that is closely associated with the incidence of CKD, ESRD, and renal failure. There is ample evidence supporting a significant relationship between obesity, CKD development, and renal failure (42–44), with those individuals exhibiting abdominal obesity facing a greater risk of CKD, potentially owing to renal sinus fat deposits. Indeed, the available evidence regarding correlations between fat deposits in the renal sinuses or other sites, particular metabolic indices, anthropometric measurements, and clinical characteristics including blood pressure suggests that renal sinus fat acts as an ectopic fat depot related to renal metabolic activity. Renal sinus fat is closely related to obesity-associated conditions including CKD, atherosclerosis, and hypertension (45–47). The Framingham trial found that those patients presenting with elevated levels of renal sinus fat were more likely to exhibit CKD and hypertension, even when controlling for BMI and visceral adipose tissue (47). Higher levels of renal sinus fat are also related to a greater risk of microalbuminuria, which is associated with greater major adverse cardiovascular event and death risk among patients experiencing albuminuria and renal impairment (48–50).

MR studies have offered clear evidence in support of a causal link of MetS with elevated CKD risk (27). Specific MetS components, in particular hypertension, have consistently been shown to be related to a greater risk of CKD incidence. In this study, 161 MetS-related SNPs were initially examined, leading to the identification of a clear positive causal link of MetS with renal failure using IVW and weighted median analytical approaches. Specific analyses of MetS components further revealed that WC and hypertension were associated with a significant increase in renal failure risk. Although prior observational studies have noted links between BG, TG, or HDL-C and the incidence of CKD or renal failure (51–55), the present approach failed to detect any significant causal association between these metabolic markers and renal failure. Overall, these data suggest that the link between MetS and renal failure may be primarily attributable to the effects of visceral obesity and hypertension, emphasizing the key role that these metabolic factors play in kidney disease pathogenesis.

In past studies, researchers have largely utilized retrospective approaches, introducing confounding variables that may have adversely impacted study results. Addressing these issues by conducting large-scale randomized clinical trials can be expensive and labor-intensive, complicating the research process. In contrast, the MR approach employed herein offers a means of readily minimizing the risk of error due to reverse causality of confounding effects, enabling more accurate and precise analyses of the links between MetS or components thereof and renal failure. Through the use of extant GWAS summary-level data, this study performed in-depth analyses of both MetS as a whole and its specific components, with corresponding sensitivity analyses providing greater credibility to these analytical results. These sensitivity analyses are vital for the identification and correction of potential errors, ensuring greater result stability and reliability. This in-depth analytical strategy yielded greater validity to the overall approach, ensuring that the causal link of MetS with renal failure could be examined in detail.

This study is subject to some limitations. The results presented herein are primarily derived from a population of European ancestry. As such, the generalizability of these findings to populations of different ancestries may be limited. The unique genetic make up, lifestyle, and environmental factors prevalent in different populations could potentially influence the relationship between MetS and kidney failure. Therefore, it is crucial to replicate these findings in diverse populations to ensure their broad applicability. In addition, no stratified or subgroup analyses were conducted in this study, potentially limiting the ability to detect causal relationships of MetS with different types of renal failure. Future research efforts are thus warranted to address these limitations, including conducting studies in diverse populations and performing stratified or subgroup analyses, in order to better understand how MetS is linked to kidney failure. In addition to the aforementioned limitations, it is important to acknowledge the assumptions inherent in MR analysis. MR relies on three core assumptions: (i) associated with the exposure (the relevance assumption); (ii) have no common cause with the outcome (the independence assumption); and (iii) have effects on the outcome solely through the exposure (the exclusion restriction assumption) (18). In MR, (i) is relatively straightforward to test, while (ii) and (iii) are difficult to establish unequivocally. As a prominent example, horizontal or type I pleiotropy has been shown to be common in genetic variation, which can bias MR estimates (56, 57). This occurs when a genetic instrument is associated with multiple traits other than the outcome of interest. To detect and correct this as best as possible, we used various MR tests as sensitivity analyses that each aim to adjust for or account for the presence of horizontal pleiotropy, including MR-PRESSO, as well as MR-Egger and weighted median methods. There is no universally accepted method that is perfectly robust to horizontal pleiotropy, but we take the best current approach by using multiple methods and examining the consistency of results.

## 5 Conclusion

In conclusion, these findings provide support for a causal association of MetS with renal failure. There is thus a clear need for clinicians to carefully monitor patients with MetS and evaluate these individuals for the risk of advancement to the state of renal failure. In CKD patients, there is a need to more effectively manage MetS, focusing in particular on those individuals with a larger WC and the management of hypertension in these patients.

## Data availability statement

The original data from the study report has been included in the article **Supplementary Materials**. For any inquiries, please contact the relevant authors. Relevant GWAS data websites: https://gwas.mrcieu.ac.uk/datasets, https://www.ebi.ac.uk/gwas, https://www.finngen.fi/en. https://cncr.nl/ctg/.

## Ethics statement

The data are sourced from public databases available from the aforementioned sources. Detailed URLs for data collection are provided in **Table 1**. All personal data were anonymized, and ethical approval and informed consent were waived.

## Author contributions

XC: Writing – review & editing, Writing – original draft, Funding acquisition, Data curation, Conceptualization. DW: Writing – original draft, Formal analysis, Data curation. JW: Writing – original draft, Project administration, Methodology, Investigation. CD: Writing – original draft, Resources, Project administration, Methodology. YL: Writing – original draft, Supervision, Software, Investigation. JZ: Writing – review & editing, Writing – original draft, Visualization, Validation, Supervision. WX: Writing – review & editing, Writing – original draft, Visualization, Validation, Supervision.
