# Supplementary figures and images for: A mendelian randomization study revealing that metabolic syndrome is causally related to renal failure
Source: Front Endocrinol (Lausanne). 2024 Jun 7;15:1392466. doi: 10.3389/fendo.2024.1392466 (PMC11190295; doi:10.3389/fendo.2024.1392466)

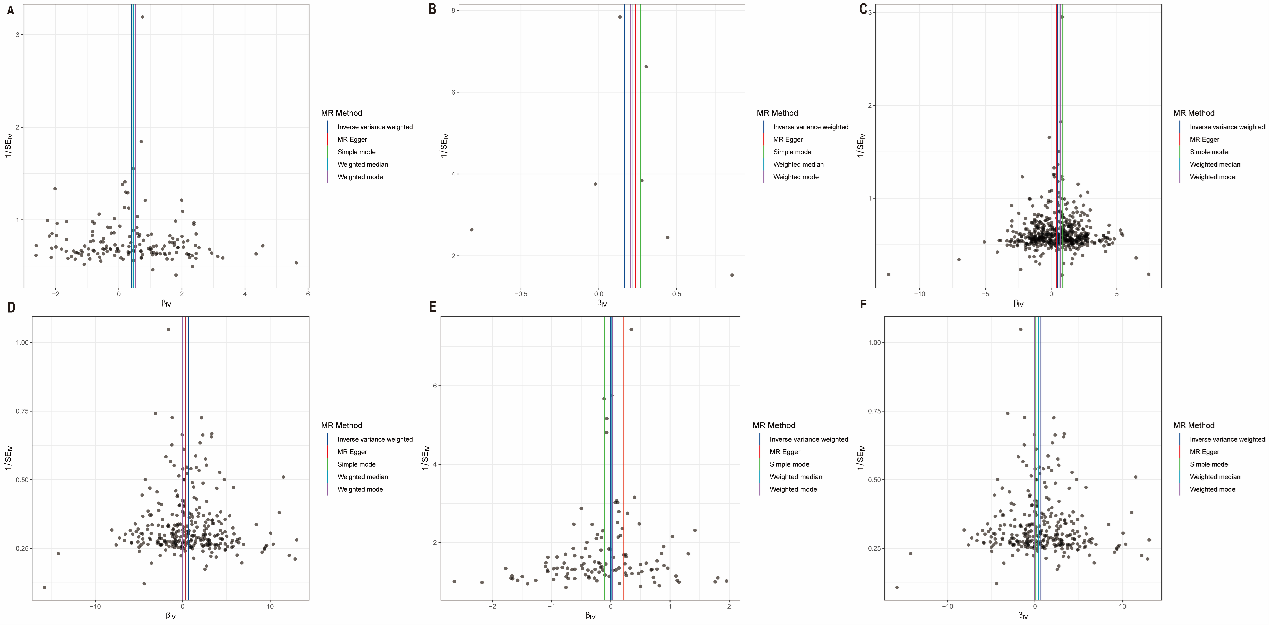

Supplement: Supplementary file 1 [file Image_1.tif]

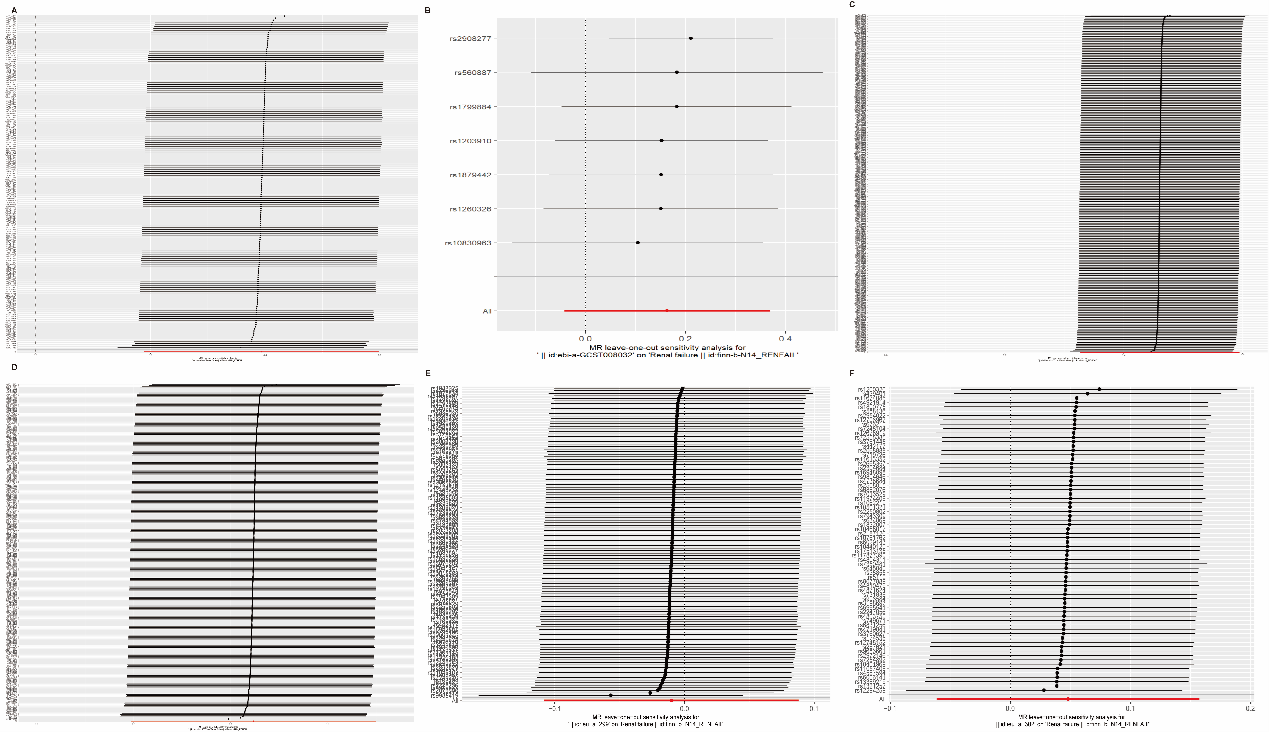

Supplement: Supplementary file 2 [file Image_2.tif]
